# Successful transcatheter edge-to-edge repair for tricuspid regurgitation in a patient with a double-inlet left ventricle and discordant connections of the great arteries: a case report

**DOI:** 10.1093/ehjcr/ytae659

**Published:** 2024-12-11

**Authors:** Masashi Yamaguchi, Takashi Matsumoto, Tomoki Ochiai, Shingo Mizuno, Shigeru Saito

**Affiliations:** Department of Cardiology and Catheterization Laboratories, Shonan Kamakura General Hospital, Okamoto 1370-1, Kamakura City, Kanagawa 247-8533, Japan; Department of Cardiology and Catheterization Laboratories, Shonan Kamakura General Hospital, Okamoto 1370-1, Kamakura City, Kanagawa 247-8533, Japan; Department of Cardiology and Catheterization Laboratories, Shonan Kamakura General Hospital, Okamoto 1370-1, Kamakura City, Kanagawa 247-8533, Japan; Department of Cardiology and Catheterization Laboratories, Shonan Kamakura General Hospital, Okamoto 1370-1, Kamakura City, Kanagawa 247-8533, Japan; Department of Cardiology and Catheterization Laboratories, Shonan Kamakura General Hospital, Okamoto 1370-1, Kamakura City, Kanagawa 247-8533, Japan

**Keywords:** Congenital heart disease, Tricuspid regurgitation, Valve repair, MitraClip, Case report

## Abstract

**Background:**

In patients with adult congenital heart disease (ACHD), significant atrioventricular valve regurgitation is an important risk factor for poor outcomes, such as heart failure. However, in many cases, transcatheter intervention may reduce the risk profile to avoid a high surgical risk.

**Case summary:**

A 44-year-old man with complex ACHD in the form of a double-inlet left ventricle, congenitally corrected transposition of the great arteries, pulmonary atresia, atrial septal defect, and patent ductus arteriosus was referred for the treatment of severe tricuspid regurgitation. He received an aortopulmonary shunt and a left-sided modified Blalock-Taussig shunt during childhood. Because of the patient’s high surgical risk due to seroma formation around the two shunts and intra-mediastinal collateral vessels, the heart team opted for transcatheter edge-to-edge repair (TEER) using a MitraClip (Abbott Vascular, Santa Clara, CA, USA). Tricuspid TEER was successfully performed using the MitraClip G4 system. The postoperative course was uneventful, with significant improvements in the New York Heart Association functional class.

**Discussion:**

Our case demonstrates that tricuspid TEER can be an alternative option for patients with complex ACHD who are at high risk for conventional surgeries; however, careful assessment with multimodality imaging and a heart team approach, including a cardiologist, ACHD specialist, cardiac surgeon, anthologist, and intensivist, should be considered.

**Learning points**

- Heart failure is common in patients with complex adult congenital heart disease (ACHD); however, these patients are generally high risk for open-heart surgery.
- Tricuspid transcatheter edge-to-edge repair conducted with off-label use of the MitraClip system may be a feasible and safe strategy for selected patients with complex ACHD.
- The heart team approach is crucial for successful management of complex haemodynamics in patients with ACHD.

## Introduction

The number of patients with adult congenital adult heart disease (ACHD) has increased in recent decades; heart failure (HF) is a serious problem in these patients, as it is associated with morbidity and mortality.^1^ Double-inlet left ventricle (DILV) is rare, with an incidence of 0.05–0.1 in every 1000 live births.^2^ The Fontan procedure, the last surgical option for patients with a single ventricle, improves survival and increases functional capacity in most cases.^3^ However, long-term outcomes of patients with a single ventricle who do not undergo the Fontan procedure include poor prognosis because of cardiovascular complications, especially arrhythmia and HF hospitalization.^4^ Atrioventricular valve (AVV) regurgitation develops insidiously during follow-up due to previous volume overload because of a systemic-to-pulmonary shunt, annular dilation, and abnormal chordae and papillary muscles.^5^ Atrioventricular valve regurgitation is associated with arrhythmia and HF; therefore, substantial AVV regurgitation should be carefully assessed using an interventional approach.

## Summary figure

**Figure:**
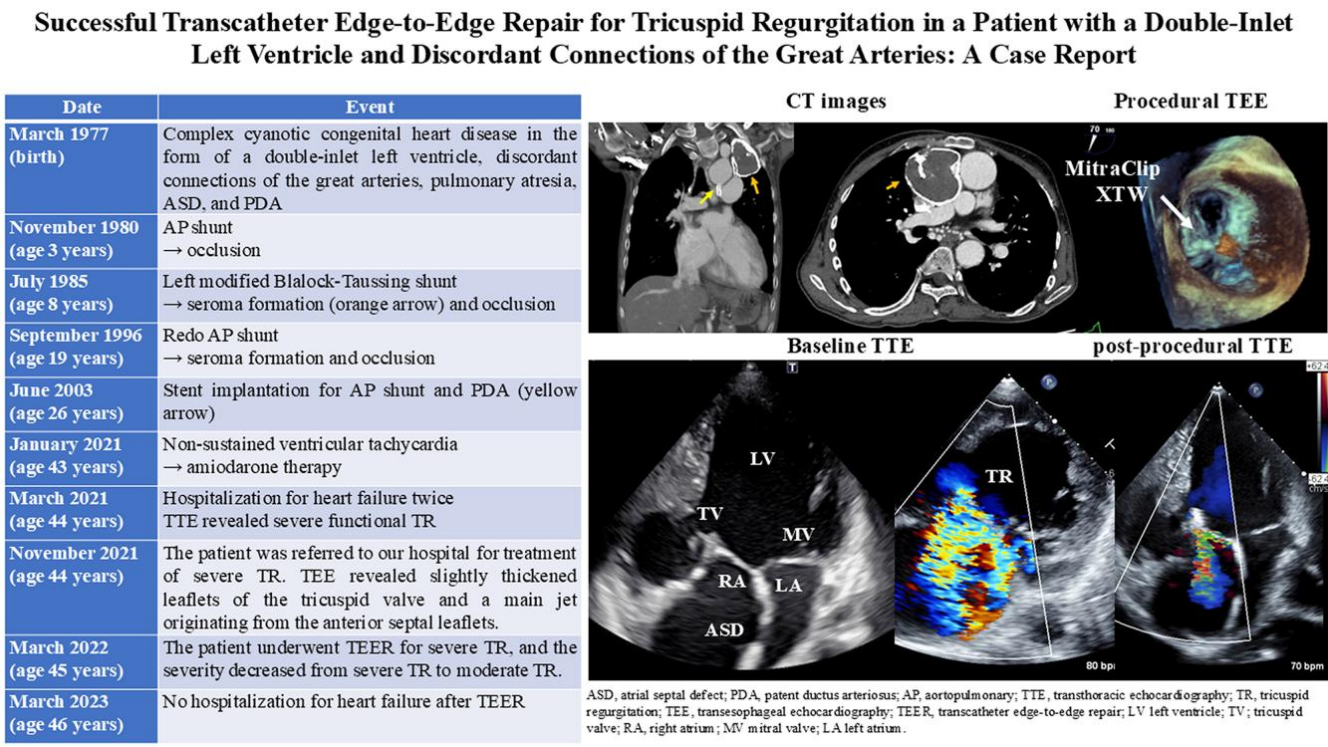


## Case presentation

**Figure 1:**
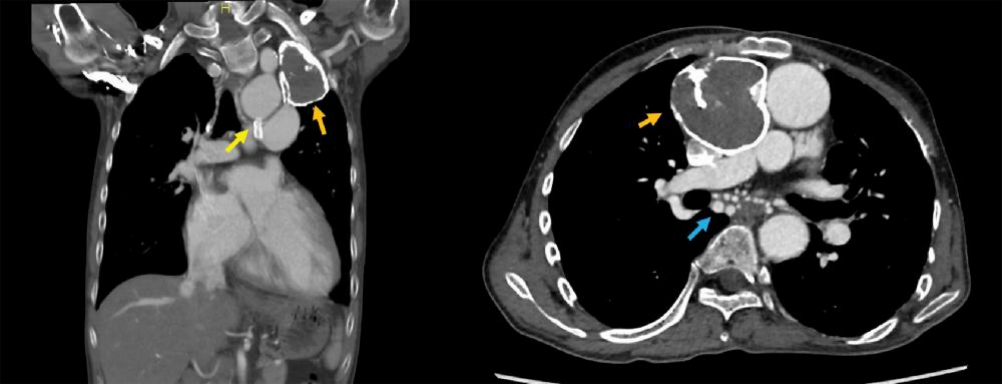
Contrast computed tomography showing a univentricular heart (double-inlet left ventricle), atrial septal defect, seroma formation (orange arrow), and patent ductus arteriosus (yellow arrow). Collateral vessel formation in the mediastinum is also increased (blue arrow).

A 44-year-old man with complex cyanotic congenital heart disease (CHD) in the form of a univentricular heart (DILV), discordant connections of the great arteries, pulmonary atresia, atrial septal defect, and patent ductus arteriosus was referred to our hospital for treatment of severe tricuspid regurgitation (TR). He received an aortopulmonary (AP) shunt and left-sided modified Blalock-Taussig shunt during childhood; however, they became occluded due to seroma formation. He underwent stent implantation for an AP shunt and patent ductus arteriosus at 26 years old (*Figure 1*). In the last 2 years, he complained of deterioration of his New York Heart Association functional class from I to III due to the development of non-sustained ventricular tachycardia and severe TR. Although the non-sustained ventricular tachycardia was successfully treated with amiodarone, his HF symptoms (stage C) remained.

On admission, a physical examination revealed the following: heart rate, 70 b.p.m.; blood pressure, 92/46 mmHg; and O_2_ saturation, ∼85%. The patient had peripheral cyanosis, finger clubbing, and a pansystolic murmur. His daily medications were enalapril 7.5 mg, spironolactone 75 mg, methyldigoxin 0.1 mg, furosemide 30 mg, tolvaptan 3.75 mg, warfarin 1 mg, and amiodarone 100 mg.

**Figure 2:**
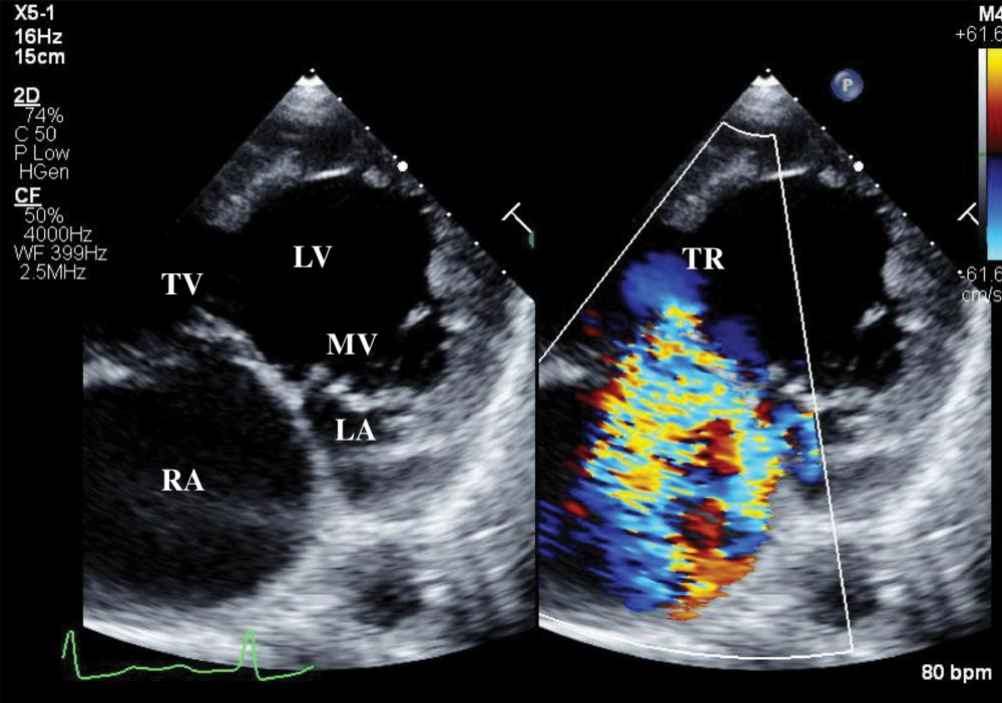
Baseline transthoracic echocardiography revealed a univentricular heart (double-inlet left ventricle) with severe tricuspid regurgitation. LV, left ventricle; TV, tricuspid valve; MV, mitral valve; RA, right atrium; LA, left atrium; TR, tricuspid regurgitation.

**Figure 3:**
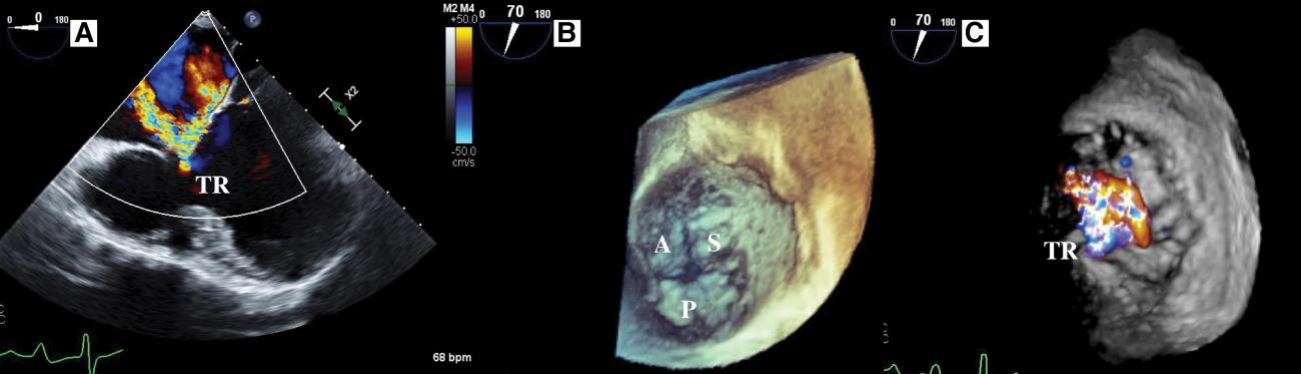
Baseline transesophageal echocardiography images. (*A*) 2D transesophageal echocardiography pre-procedural imaging demonstrates severe tricuspid regurgitation. (*B*) 3D transesophageal echocardiography imaging shows poor tricuspid valve coaptation between the anterior and septal tricuspid leaflets. (*C*) Severe tricuspid regurgitation originating from the anterior/septal tricuspid leaflets. TEE, transesophageal echocardiography; TR, tricuspid regurgitation; A, anterior tricuspid leaflet; S, septal tricuspid leaflet; P, posterior tricuspid leaflet.

Blood tests revealed a B-type natriuretic peptide level of 138.1 (reference, 0–18.4) pg/mL, normal creatinine level (87.5; reference, 57.5–94.6 μmol/L), and no anaemia (haemoglobin, 17.9; reference, 13.7–16.8 g/dL). Chest radiography did not reveal pulmonary oedema or pleural effusion. Electrocardiography revealed a sinus rhythm without any specific intraventricular conduction delay or T-wave changes. Transthoracic echocardiography (TTE) revealed moderate systolic ventricular dysfunction (end-diastolic volume, 254.0 mL; end-systolic volume, 127.0 mL; ejection fraction, 39.7%) with severe TR (elective regurgitant orifice, 0.31 cm^2^; regurgitant volume, 50 mL/beat; vena contract, 6.7 mm) (*Figure 2*; Supplementary material online, *Video S1*). Transesophageal echocardiography (TEE) showed slightly thickened leaflets of the tricuspid valve and a main jet originating from the anterior septal leaflets due to annular dilatation and leaflet tethering (*Figure 3*). Invasive heart catheterization did not reveal elevated right or left atrial pressure; however, the pulmonary artery pressure was relatively high (mean right atrium, 8 mmHg; mean left atrium, 8 mmHg; pulmonary artery 21/15, mean, 18 mmHg; mean pulmonary capillary wedge pressure, 10 mmHg).

Patients with complex CHD develop symptoms of HF with multifactorial causes. Our patient complained of fatigue and dyspnoea on exertion with exacerbation of ventricular arrhythmia and valvular heart disease. Even after successful management of his non-sustained ventricular tachycardia, his symptoms remained unchanged. Therefore, the most probable diagnosis was low-output syndrome caused by severe TR. Although tricuspid valve replacement and Glenn surgery were considered as treatment options in the ACHD department of a previous hospital, they were deemed too high risk because of the patient’s history of two thoracotomies and increased collateral vessel formation in the mediastinum (*Figure 1*). Therefore, transcatheter edge-to-edge repair (TEER) with a MitraClip (Abbott Vascular, Santa Clara, CA, USA) for TR followed by staged Glenn surgery was chosen after discussions between the heart teams of the previous hospital and our hospital. One concern is the technical complexity of tricuspid TEER in patients with ACHD. Despite the patient’s complex haemodynamics, computed tomography revealed no abnormalities at the access site from the femoral vein to the tricuspid valve. Therefore, tricuspid TEER was considered feasible from a technical perspective.

**Figure 4:**
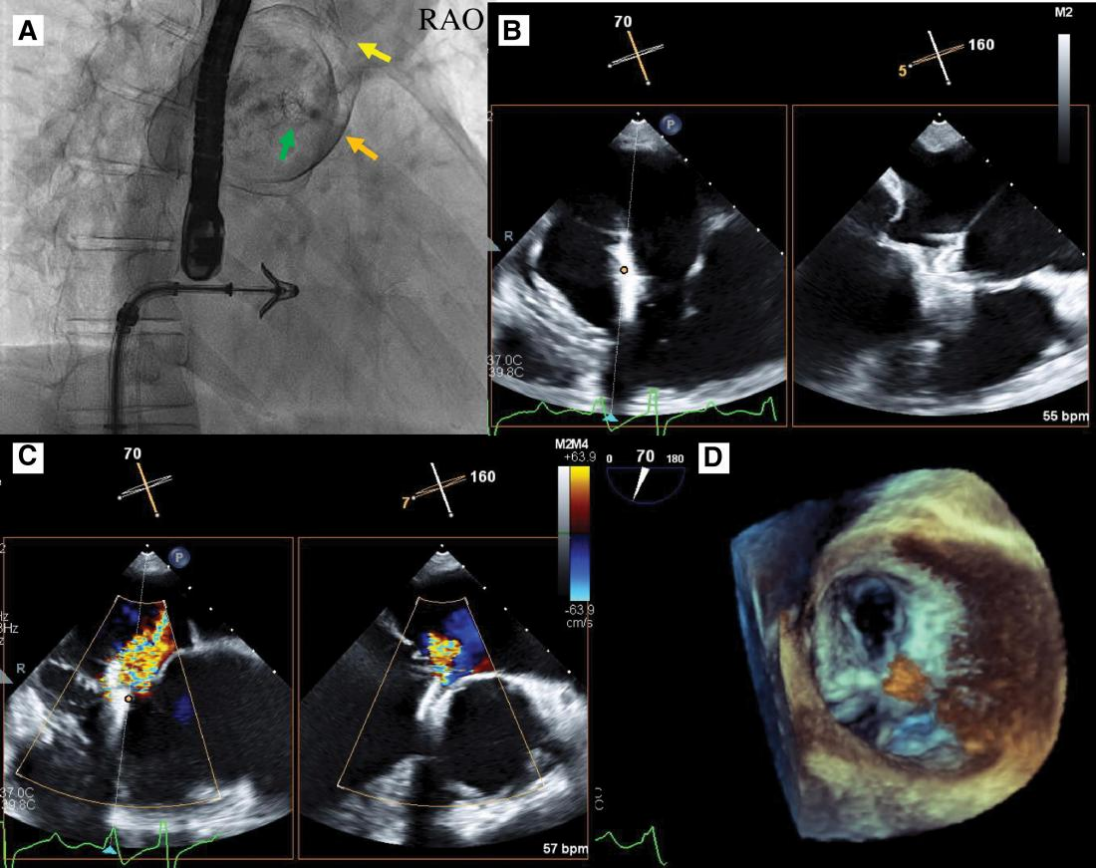
Procedural transesophageal echocardiography and fluoroscopic images. (*A*) Fluoroscopic image shows the MitraClip in a straddling position and stent implantation of an arterioportal shunt (green arrow) and patent ductus arteriosus (yellow arrow) with seroma formation (orange arrow). (*B*) Transesophageal echocardiography demonstrating an attempt to grasp two leaflets with the clip. (*C*) Post-procedural moderate tricuspid regurgitation. (*D*) Double-orifice tricuspid valve after MitraClip insertion. TEE, transesophageal echocardiography; RAO, right anterior oblique.

Tricuspid TEER was performed under general anaesthesia and TEE guidance. The left femoral vein was selected as the access site to gain height above the tricuspid valve. The guide catheter was smoothly advanced into the right atrium. MitraClip G4 XTW was selected because both leaflets had sufficient length to be grasped. With careful TEE assessment, the MitraClip G4 XTW was successfully deployed at the anterior septal leaflets without any complications. Following one-clip deployment, the severity of the TR decreased from 4+ (severe) to 2+ (moderate) (*Figure 4*). The mean gradient across the tricuspid valve was 3 mmHg. The patient’s haemodynamics remained stable during the procedure.

**Figure 5:**
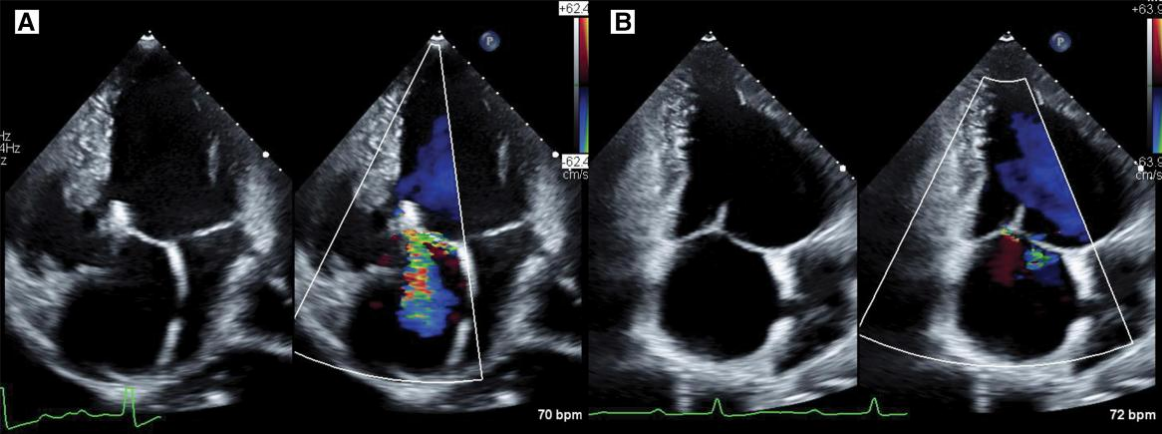
(*A*) Transthoracic echocardiography at discharge shows moderate tricuspid regurgitation. (*B*) Transthoracic echocardiography at the 1-month follow-up shows a decrease in the residual tricuspid regurgitation. TTE, transthoracic echocardiography; TR, tricuspid regurgitation.

The patient was transferred to the intensive care unit, and his postoperative course was uneventful. His blood pressure and O_2_ saturation were 100/60 mmHg and ∼85%, respectively; neither was significantly different from the preoperative values. On post-procedural Day 1, TTE showed moderate TR, and the patient was transferred to the general ward (*Figure 5A*). Right heart catheterization on post-procedural Day 3 demonstrated almost normal left atrial and pulmonary artery pressures (mean left atrium, 11 mmHg; mean right atrium, 13 mmHg; mean pulmonary artery, 18 mmHg; right ventricular pressure, 102/2, right ventricular end-diastolic pressure, 10 mmHg). The patient was discharged 4 days after the procedure, with an improvement in clinical status. The patient’s NYHA class improved from III to approximately I following the procedure. Transthoracic echocardiography at the 1-month follow-up revealed trivial TR (*Figure 5B*; Supplementary material online, *Video S2*).

## Discussion

This report described the successful use of tricuspid TEER for severe TR in a patient with a DILV and discordant connections of the great arteries. Based on the high-risk profile for open-heart surgery, severe TR was successfully managed with tricuspid TEER and off-label use of the MitraClip system.

The development of HF in ACHD has several causes, including arrhythmia, shunts, valvular heart diseases, and ventricular dysfunction (diastolic and/or systolic). Open-heart surgery is a treatment option; however, as in our case, most patients with ACHD have a high surgical risk owing to histories of open-heart surgery and/or thoracotomies, and complex haemodynamics.^6,7^ Therefore, transcatheter therapy, which is a less invasive approach, represents a promising treatment option.

Two important points explain the procedural success of the present case. The first involves features of the tricuspid valve. Tricuspid valve leaflets are generally thin and move in a flattening motion; therefore, grasping the leaflets during TEER is technically difficult. However, the leaflets in our case were thick and moved briskly, similar to mitral valve leaflets. We speculate that the pressure of a single ventricle, which is the same as the left ventricular pressure, may have changed the motion of the leaflets and thickened them. The second is the choice of access route. Although our patient had a complex ACHD, no abnormalities were encountered in the access route from the femoral vein to the tricuspid valve. Additionally, the right atrium was enlarged, making system manipulation easier.

Although the TR was effectively managed, further considerations for this case included other treatments, complications, and long-term management owing to the patient’s young age. The rate of sudden cardiac death in patients with ACHD is up to 100 times more frequent than in patients with acquired heart disease.^8^ Additionally, 7.5% of patients with a single ventricle who do not undergo Fontan repair undergo heart transplantation, and 2.5% have an implantable cardioverter-defibrillator (ICD).^9^ Given the severe long-term effects of amiodarone, which can lead to organ toxicities and increase the risk of sudden death, we are considering ICD implantation for prevention of sudden cardiac death. Other treatments, such as ventricular septation and Fontan repair, were also considered. However, because of the high risk associated with these procedures, their indications and strategies should be meticulously reviewed. As a last resort, heart transplantation could be considered. It is important to provide comprehensive information to patients and their family well in advance, ensuring they are fully informed about the potential risks and benefits of each treatment.

Although limited therapeutic options are available, our case shows that tricuspid TEER with off-label use of the MitraClip system is a possible therapeutic option for TR in selected patients with complex ACHD. Nevertheless, evidence-based treatment options are required to improve patient safety and prognosis.

## Supplementary Material

ytae659_Supplementary_Data

## Data Availability

The data sets used and/or analysed during the current study are available from the corresponding author on reasonable request.

## Lead author biography

Masashi Yamaguchi has been working at Shonan Kamakura General Hospital since April 2020. He graduated from Miyazaki University in 2009. His primary areas of interest include structural heart interventions. He is a member of the Japanese Circulation Society, Japanese Association of Cardiovascular Intervention and Therapeutics, and Japanese Transcatheter Valve Therapy.

## Notes

**Consent:** Informed consent was obtained from the patient in accordance with the Committee on Publication Ethics (COPE) guidelines.

**Funding:** None declared.
